# Delayed Numerical Chromosome Aberrations in Human Fibroblasts by Low Dose of Radiation

**DOI:** 10.3390/ijerph121214979

**Published:** 2015-12-01

**Authors:** Yoon Hee Cho, Su Young Kim, Hae Dong Woo, Yang Jee Kim, Sung Whan Ha, Hai Won Chung

**Affiliations:** 1 Department of Molecular Epidemiology, School of Public Health, Seoul National University, 1 Gwanak-ro, Gwanak-gu, Seoul 151-742, Korea; chugnhw@snu.ac.kr; 2 Department of Preventive Medicine, School of Medicine, Jeju National University, 66 Jejudaehakno, Jeju-si, Jeju-do 690-756, Korea; suy0202@jejunu.ac.kr; 3 Molecular Epidemiology Branch, Division of Cancer Epidemiology and Prevention, Research Institute, National Cancer Center, Goyang-si, Gyeonggi-do 410-769, Korea; eastsea93@hanmail.net; 4 Da Vinci College of General Education, Chung-ang University, 84 HeukSeok-Ro, DongJak-gu, Seoul 156-756, Korea; yangjee4@hanmail.net; 5 Department of Radiation Oncology, Seoul National University College of Medicine, 101 Daehak-ro, Jongno-gu, Seoul 110-744, Korea; swha@snu.ac.kr; 6 Current address: Department of Biomedical and Pharmaceutical Sciences, College of Health Professions and Biomedical Sciences, University of Montana, 32 Campus Drive, Missoula, MT 59812, USA

**Keywords:** radiation-induced genomic instability, X-irradiation, micronucleus-centromere assay, micronuclei, aneuploidy

## Abstract

Radiation-induced genomic instability refers to a type of damage transmitted over many generations following irradiation. This delayed impact of radiation exposure may pose a high risk to human health and increases concern over the dose limit of radiation exposure for both the public and radiation workers. Therefore, the development of additional biomarkers is still needed for the detection of delayed responses following low doses of radiation exposure. In this study, we examined the effect of X-irradiation on delayed induction of numerical chromosomal aberrations in normal human fibroblasts irradiated with 20, 50 and 100 cGy of X-rays using the micronucleus-centromere assay. Frequencies of centromere negative- and positive-micronuclei, and aneuploidy of chromosome 1 and 4 were analyzed in the surviving cells at 28, 88 and 240 h after X-irradiation. X-irradiation increased the frequency of micronuclei (MN) in a dose-dependent manner in the cells at all measured time-points, but no significant differences in MN frequency among cell passages were observed. Aneuploid frequency of chromosomes 1 and 4 increased with radiation doses, and a significantly higher frequency of aneuploidy was observed in the surviving cells analyzed at 240 h compared to 28 h. These results indicate that low-dose of X-irradiation can induce delayed aneuploidy of chromosomes 1 and 4 in normal fibroblasts.

## 1. Introduction

Ionizing radiation can induce a large spectrum of DNA lesions directly by energy absorption and indirectly by production of reactive free radicals. These lesions include single-strand DNA breakages, double-strand DNA breakages (DSBs), DNA base alterations and DNA-DNA crosslinks [1]. Genomic instability, defined as “the increased rate of acquisition of alterations in the genome” [2], is a driving force underlying radiation carcinogenesis [3]. The general belief is that genomic instability induced by radiation is due to the DNA being changed immediately after exposure, during the repair of DNA damage, or during DNA replication [4]. However, a new paradigm of radiation biology suggests that the initial lesions caused by radiation exposure in cellular DNA can also be transmitted from the surviving exposed cell through multiple cell generations to be expressed in the progeny of surviving cells [5]. In addition, several experiments have shown that delayed and non-targeted genomic instability may arise with bystander effects, where communication between irradiated and un-irradiated cells leads to the induction of instability [2,6,7,8,9]. This delayed and non-targeted effect of radiation exposure may pose a high risk to human health and raises concerns regarding the dose limit of radiation exposure for both the public and radiation workers.

The biological effects of radiation exposure at low doses are still uncertain, which complicates the study of any associated health effects [10]. Furthermore, radiation-induced genomic instability (RIGI) may play an important biological role in low-dose radiation exposures. However, the concept of RIGI, including delayed and non-targeted effects are mostly based on findings obtained using high linear energy transfer (LET) charged-particle radiation [11,12,13,14] and include very few studies using low-LET radiation [15,16]. Elucidating the biological effects of low-LET and low-dose ionizing radiation has important implications in ensuring radiation safety and protection. Therefore, there is a critical need for the development of additional biological markers that will enable the detection of delayed responses following exposure to low-LET radiation at low doses.

Genomic instability in general can be manifested by a variety of cellular changes and a variety of endpoints have been used to describe RIGI, such as decreased plating efficiency, micronuclei (MN) formation, increased sister chromatid exchange and chromosomal instability [2,6]. Chromosomal instability is a particularly striking form of general instability and it may play an important role in the early stages of cancer progression [17]. The most notable types of chromosomal instability in the cell genome involve aneuploidy [18], gene deletion [19] and both “stable” and “unstable” chromosome aberrations [2,6].

In particular, aneuploidy resulting from the errors in mitotic chromosome segregation is the most common genetic alteration observed in human tumors [20,21]. Several studies reported that mutations in genes encoding major mitotic proteins or chromosome segregation proteins could induce aneuploidy [22,23,24]. Although the molecular basis responsible for aneuploidy during carcinogenesis is still undefined [25], increased aneuploidy is known to play a critical role in genomic instability and the development of cancer [26,27,28]. Furthermore, to our knowledge, very few (if any) studies have assessed the delayed effect of low-dose of radiation on aneuploidy, although several studies have reported delayed RIGI [7,8].

Therefore, we aimed to examine whether low-dose of X-irradiation can induce delayed chromosomal instability in normal human fibroblasts. To this end, normal human fibroblasts were irradiated with three different doses (20, 50 and 100 cGy) of X-ray then sub-cultured up to five passages. We performed the micronucleus-centromere assay with chromosome-specific composite DNA and/or pan-centromeric probes, which facilitated an accurate analysis of radiation-induced structural breaks and numerical chromosome rearrangements including non-disjunction and chromosome loss [29]. Using this assay, the MN derived from acentric chromosome fragments can be distinguished from whole chromosome-derived MN [30,31]. Chromosomes 1 and 4 were selected for analysis of aneuploidy in this study since they were more sensitive to irradiation than other chromosomes [32,33].

## 2. Experimental Section

### 2.1. Cell Culture and Irradiation

Normal human fibroblasts (CCD-986sk) were obtained from the Korean Cell Line Bank. The cells had a normal karyotype, and were maintained in a monolayer culture in AminoMAX^TM^-C100 basal medium with AminoMAX^TM^-C100 supplement (Gibco, Grand Island, NY). The cells were seeded in T25 flasks (25 cm^2^) and cultured at 37 °C in a humidified incubator with 5% CO_2_ in air. As the doubling time of the cells was around 40 h, the cells were subcultured every 36–40 h to maintain exponential growth.

The human fibroblasts cells were X-irradiated using a LINAC (Varian 6/100, Palo Alto, CA) with doses of 20, 50 or 100 cGy (dose rate: 200 cGy/min). After irradiation, the cells were washed twice with phosphate-buffered saline (PBS), and further incubated for 28, 88 (1 passage), and 240 h (5 passages). The cells were sub-cultured at the population doubling level, thus 88 and 240 h culturing conditions are equivalent to 1 and 5 population doublings.

### 2.2. The Cytokinesis-Block Micronucleus Assay

To evaluate the direct effect of X-ray irradiation, cytochalasin-B (Sigma, St. Louis, MO, USA) was added to irradiated cells at a final concentration of 1.5 μg/mL after washing twice with PBS; this was followed by another incubation for 28 h before harvesting.

To investigate delayed effects of X-irradiation, the cells were incubated for 88 and 240 h; cytochalasin-B was added 28 h before harvest. Cells were trypsinized from confluent cultures, resuspended in complete medium then subjected to hypotonic conditions with 0.075 M KCl and fixed with methanol/acetic acid (3:1).

### 2.3. Fluorescent *in Situ* Hybridization (FISH) Using Pan-Centromeric Probe

To detect the centromere negative- and positive-micronuclei (MNC− and MNC+), FISH using a human pan-centromeric probe directly labeled with fluorescein isothiocyanate (FITC) (Cambio, Cambridge, UK) was performed according to the manufacturer’s instructions. Slides were pre-treated with proteinase K, then denatured in 70% (*v/v*) formamide, 2× Saline-Sodium Citrate (SSC, Amresco, Solon, OH, USA) solution for 2 min (pH 7.3) at 72 °C and subsequently dehydrated in graded 70%, 85%, and 100% (*v/v*) ethanol. The DNA probe was denatured at 85 °C for 10 min, applied to each slide, then cover slipped, sealed, and incubated in a humidified chamber. Following an overnight hybridization at 42 °C, slides were washed in 50% formamide/2× SSC for 5 min two times for 37 °C. Signals of the FITC hybridized probes were then amplified and counterstained with a FITC Amplification Kit (Cambio).

### 2.4. FISH Using Centromeric Probes of Chromosomes 1 and 4

To detect the aneuploidy of chromosome 1 and 4, FISH was performed using a direct-labeled centromeric probe (Vysis, Downer Grove, IL) according to the manufacturer’s instructions. We used direct-labeled centromeric probes conjugated with Spectrum Orange fluorophores (chromosome 1) and Spectrum Green fluorophores (chromosome 4). 4′,6′-diamidino-2-phenylindole (DAPI, Vysis) was used in the anti-fade solution of p-phenylenediamine dihydrochloride/glycerol for counterstaining. Briefly, the slides were denatured using 70% (*v/v*) formamide in 2 × SSC solution (pH 7.3) at 74 °C for 5 min and subsequently dehydrated in graded 70%, 85%, and 100% (*v/v*) ethanol. The DNA probes were denatured at 73 °C for 5 min and applied to each slide, which were then coverslipped, sealed, and incubated overnight at 37 °C in a humidified chamber. The slides were washed in 0.4 × SSC (pH 7.0) at 71 °C for 2 min, followed by washing in 2 × SSC/0.1% (*v/v*) NP-40 (Vysis) at 37 °C for 1 min. Finally, the slides were counterstained with DAPI and stored at −20 °C.

### 2.5. Microscopic Examination

The hybridized slides were randomized, scored blindly and viewed using a fluorescent microscope equipped with an epifluorescent illuminator at a magnification of 100 × with the numerical aperture set at 1.4 (Nikon, Tokyo, Japan). The triple band-pass filter for DAPI/FITC/Texas Red (Chroma Technology Corp., Brattleboro, VT) was used to visualize the markers. For each slide, 1000 binucleated (BN) cells were scored. For the evaluation of MN and aneuploidy, the criteria of Fenech [34] and Sgura *et al.* [35] were applied, respectively, and MNC+ and MNC− were determined by detecting the presence or absence of centromeric signals. MNC+ contained single or multiple signal(s) from all chromosomes, although we were unable to identify the individual chromosomes.

### 2.6. Statistical Analysis

Statistical analysis was performed using the SPSS 18.0 for Windows statistical package (SPSS Inc., Chicago, IL, USA). All experiments were performed twice and inter-group differences between two experiments were tested by analysis of variance (ANOVA). ANOVA testing showed no inter-group difference between each experiment (*p* > 0.05). The association of abnormality yields including MN, MNC+, MNC−, and aneuploidy of chromosomes 1 and 4 with radiation dose was tested by the Kendall rank correlation coefficient (τ). The Mann–Whitney nonparametric test was used to investigate differences among the frequencies of MN and aneuploidy in cells of different passages. The criterion for significance was set at *p* < 0.05.

## 3. Results

### 3.1. Radiation Increased the Frequency of MN, but not Delayed MN Induction

As shown in Table 1, X-irradiation significantly increased frequencies of MN in a dose-dependent manner (*p* < 0.001), with mean MN frequencies of 30.5 ± 2.1, 37 ± 2.8, 65 ± 2.1 and 113 ± 7.0 per 1000 BN cells at 0, 20, 50 and 100 cGy, respectively. MN frequencies in the cells analyzed at 88 and 240 h were also examined; the level of MN at 240 h was not significantly different from either the level at 28 or 88 h (*p* > 0.05).

**Table 1:** The frequencies of micronuclei in the X-irradiated fibroblasts at all time points.

Time after Irradiation (h)	Dose (cGy)	No. of MNCB *^a^* Cells/1000 BN *^b^* Cells	Multi MNCB *^c^*/MNCB Cells (%) ^†^	Total No. of MN *^d^*/1000 BN Cells
28 (Direct)	Control	28.0 ± 1.4	9.8 ± 2.5	30.5 ± 2.1
	20	35.0 ± 0.0	6.1 ± 2.2	37.0 ± 2.8
	50	55.5 ± 0.7	19.1 ± 3.4	65.0 ± 2.1
	100	95.5 ± 3.5	18.9 ± 1.4	113.0 ± 7.0 *
88 (1 passage)	Control	30.0 ± 0.0	5.3 ± 1.6	31.5 ± 0.7
	20	33.0 ± 0.0	6.5 ± 1.7	35.5 ± 2.1
	50	57.0 ± 2.8	9.6 ± 1.4	62.0 ± 5.6
	100	99.5 ± 0.7	13.8 ± 3.5	114.5 ± 3.5 *
240 (5 passages)	Control	31.0 ± 2.8	5.1 ± 1.9	32.5 ± 2.1
	20	33.5 ± 2.1	9.8 ± 2.8	37.0 ± 2.8
	50	58.0 ± 4.2	9.4 ± 1.5	64.0 ± 8.4
	100	101.5 ± 0.7	8.0 ± 4.2	111.0 ± 4.2 *

The numbers indicate the mean of two experiments ± standard deviation (SD) of the mean. *^a^* Micronucleated cytokinesis-blocked; *^b^* Binucleated; *^c^* Cells with several micronuclei; *^d^* Micronuclei. ^†^ The ratios were calculated from individual values. * Significantly increased with radiation dose by Kendall’s τ calculated on cell bases.

### 3.2. Induction of MNC+ and MNC− Did Not Differ with Number of Cell Passages After X-Irradiation

**Figure 1:**
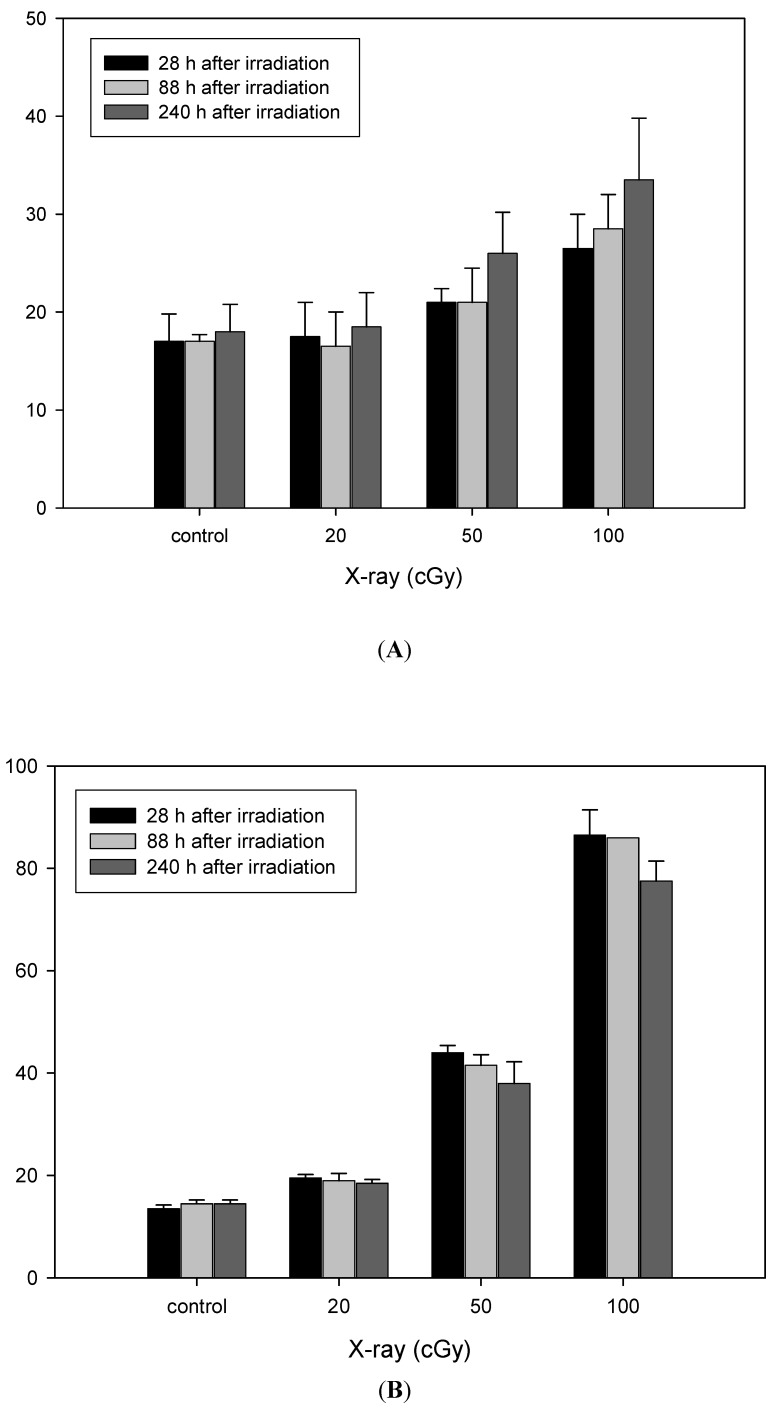
The frequencies of (**A**) MNC+ and (**B**) MNC− in X-irradiated fibroblasts. Data are the mean ± SD of duplicate experiments.

As shown in Figure 1, frequencies of MNC+ and MNC− were significantly increased with radiation dose after X-irradiation (*p* < 0.05). The pan-centromeric probe-based FISH revealed that >70% of MN were MNC− after X-irradiation. There were no significant differences in either MNC+ or MNC− frequencies with cell passage after cells were exposed to 20 cGy.

MNC− frequency at 88 and 240 h was decreased in comparison to 28 h after 50 and 100 cGy of X-irradiation; however, this decrease was not statistically significant (*p* > 0.05). In contrast to MNC−, the induction of MNC+ after 50 and 100 cGy of X-irradiation showed a tendency for incremental increases with cell passage, but this was also not statistically significant (*p* > 0.05).

### 3.3. Radiation Induced Delayed Aneuploidy of Chromosomes 1 and 4 in Fibroblasts

**Figure 2:**
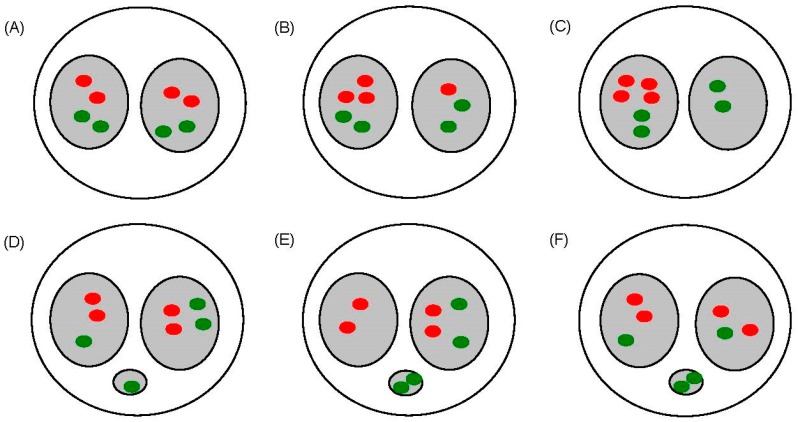
Schematic of FISH signals to demonstrate aneuploidy of chromosomes 1 (red) and 4 (green). (**A**) Normal cell with two signals in each daughter nucleus. (**B**,**C**) Aneuploidy induced by non-disjunction. No signal was observed in micronuclei; this includes 3 + 1 (three signals in one daughter nucleus and one signal in another daughter nucleus) and 4 + 0 (four signals in one daughter nucleus only). (**D**–**F**) Aneuploidy induced by chromosome loss. Signal(s) was/were observed in micronuclei; this includes 2 +1 + 1 two signals in one daughter nucleus and one signal in another daughter nucleus plus a micronucleus with a positive signal), 2 + 0 + 2 (two signals in one daughter nucleus plus micronucleus with two positive signals) and 1 + 1 + 2 (signal in each daughter nucleus plus micronucleus with two positive signals).

Figure 2 shows various types of aneuploidy of chromosomes 1 and 4 in binucleated cells, which were examined in the study.

Table 2 and Table 3 show the frequency of aneuploidy induced by both non-disjunction and chromosome loss after X-irradiation. Aneuploidy of chromosomes 1 and 4 increased in a dose-dependent manner at all time points (*p* < 0.001). Aneuploidy of both chromosomes 1 and 4 increased with cell passage after X-irradiation. The frequency of aneuploidy in chromosomes 1 and 4 after 50 cGy of X-irradiation was 6.5 ± 2.1 and 4.5 ± 2.1 at 28 h, 13.0 ± 1.4 and 8.5 ± 0.7 at 240 h, and after 100 cGy of X-irradiation was 10.0 ± 2.8 and 6.5 ± 2.1 at 28 h, 21.0 ± 2.1 and 12 ± 1.4 at 240 h. There was a significant difference in aneuploid frequency between the cells analyzed at 28 and 240 h after 100 cGy of X-irradiation in chromosome 1 (*p* < 0.05) (Table 2 and Table 3).

**Table 2:** The frequency of different types of aneuploidy in chromosome 1 from X-irradiated fibroblasts.

Time after Irradiation (h)	Dose (cGy)	No. of BN *^a^* Cells Scored	Normal Cells *^b^*	Aneuploid Cells		Total Aneuploidy
				Non-Disjunction ^c^	Chromosome Loss ^d^	
28 (Direct)	Control	1000	997.0 ± 0.0	2.5 ± 0.7	0.5 ± 0.7	3.0 ± 0.0
	20	1000	996.5 ± 0.7	3.0 ± 1.4	0.5 ± 0.7	3.5 ± 0.7
	50	1000	993.5 ± 2.1	5.0 ± 1.4	1.5 ± 0.7	6.5 ± 2.1
	100	1000	990.0 ± 4.2	7.5 ± 0.7	2.5 ± 2.1	10.0 ± 2.8 *
88 (1 passage)	Control	1000	997.0 ± 1.4	3.0 ± 1.4	0.0 ± 0.0	3.0 ± 1.4
	20	1000	996.0 ± 0.0	3.5 ± 0.7	0.5 ± 0.7	4.0 ± 0.0
	50	1000	995.0 ± 1.4	4.0 ± 1.4	1.5 ± 0.7	5.0 ± 1.4
	100	1000	991.5 ± 2.1	7.0 ± 2.8	1.5 ± 0.7	8.5 ± 2.1 *
240 (5 passages)	Control	1000	997.0 ± 0.0	2.5 ± 0.7	0.5 ± 0.7	3.0 ± 0.0
	20	1000	992.5 ± 0.7	6.0 ± 0.0	1.5 ± 0.7	7.5 ± 0.7
	50	1000	987.0 ± 1.4	11.0 ± 0.0	2.0 ± 1.4	13.0 ± 1.4
	100	1000	979.5 ± 2.1	15.0 ± 0.0	5.5 ± 2.1	21.0 ± 2.1 *^†^

The numbers indicate the mean of two experiments ± SD of the mean. *^a^* Binucleated; *^b^* Two signals in each daughter nucleus; *^c^* Aneuploidy induced by non-disjunction. No signal was observed in micronuclei; this includes 3 + 1 and 4 + 0; *^d^* Aneuploidy induced by chromosome loss. Signal(s) was/were observed in micronuclei; this includes 2 + 1 + 1, 2 + 0 + 2 and 1 + 1 + 2. * Significantly increased with radiation dose by Kendall’s τ calculated on cell bases. ^†^ Significantly different from the cells analyzed at 28 h by Mann–Whitney on the significance level of *p* < 0.05.

**Table 3:** The frequency of different types of aneuploidy in chromosome 4 from X-irradiated fibroblasts.

Time after Irradiation (h)	Dose (cGy)	No. of BN *^a^* Cells Scored	Normal Cells *^b^*	Aneuploid Cells		Total Aneuploidy
				Non-Disjunction *^c^*	Chromosome Loss *^d^*	
28 (Direct)	Control	1000	997.5 ± 0.7	2.5 ± 0.7	0.0 ± 0.0	2.5 ± 0.7
	20	1000	997.0 ± 1.4	2.5 ± 0.7	0.5 ± 0.7	3.0 ± 1.4
	50	1000	995.5 ± 2.1	3.5 ± 0.7	1.0 ± 1.4	4.5 ± 2.1
	100	1000	993.5 ± 2.1	5.0 ± 1.4	1.5 ± 0.7	6.5 ± 2.1 *
88 (1 passage)	Control	1000	997.0 ± 1.4	2.5 ± 0.7	0.5 ± 0.7	3.0 ± 1.4
	20	1000	998.0 ± 1.4	2.0 ± 1.4	0.0 ± 0.0	2.0 ± 1.4
	50	1000	996.0 ± 1.4	4.0 ± 1.4	0.0 ± 0.0	4.0 ± 1.4
	100	1000	993.0 ± 1.4	5.0 ± 1.4	2.0 ± 0.0	7.0 ± 1.4 *
240 (5 passages)	Control	1000	997.0 ± 0.0	3.0 ± 0.0	0.0 ± 00	3.0 ± 0.0
	20	1000	995.5 ± 0.7	3.0 ± 1.4	1.5 ± 0.7	4.5 ± 0.7
	50	1000	991.5 ± 0.7	6.5 ± 0.7	2.0 ± 0.0	8.5 ± 0.7
	100	1000	988.0 ± 1.4	8.5 ± 1.4	3.5 ± 0.7	12.0 ± 1.4 *

The numbers indicate the mean of two experiments ± SD of the mean. *^a^* Binucleated; *^b^* Two signals in each daughter nucleus; *^c^* Aneuploidy induced by non-disjunction. No signal was observed in micronuclei; this includes 3 + 1 and 4 + 0; *^d^* Aneuploidy induced by chromosome loss. Signal(s) was/were observed in micronuclei; this includes 2 + 1 + 1, 2 + 0 + 2 and 1 + 1 + 2; * Significantly increased with radiation dose by Kendall’s τ calculated on cell bases.

**Figure 3:**
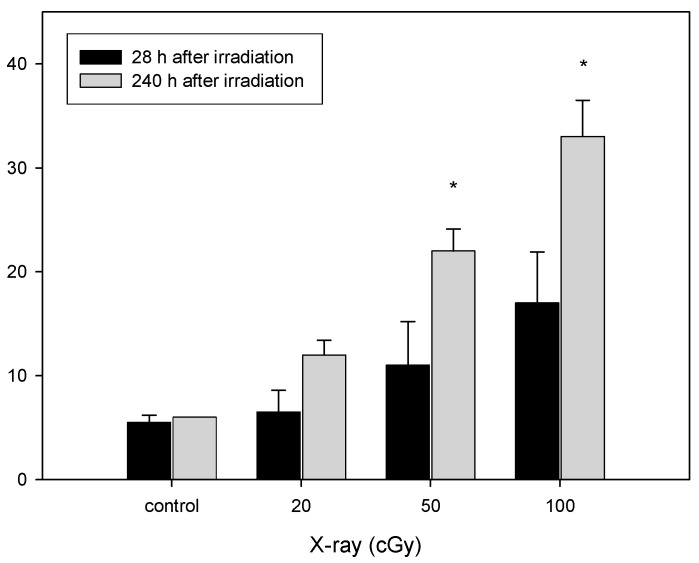
Total aneuploidy of chromosomes 1 and 4 in the X-irradiated fibroblasts. Data are the mean ± SD of duplicate experiments. Asterisks indicate significantly higher frequency of aneuploidy in chromosomes 1 and 4 from the cells at 240 h compared to 28 h (Mann–Whitney test, * *p* < 0.05).

The frequency of aneuploidy within chromosomes 1 and 4 was analyzed within a total of 1000 BN cells. Figure 3 shows total aneuploidy of chromosomes 1 and 4 induced by X-irradiation. Frequencies of total aneuploidy were 11 ± 4.2 and 16.5 ± 4.9 in the cells analyzed at 28 h, and 21.5 ± 2.1 and 33 ± 3.5 in the cells analyzed at 240 h by following 50 and 100 cGy of radiation, respectively. Significantly higher frequencies of aneuploidy were observed in the cells analyzed at 240 h compared to the cells examined at 28 h (*p* < 0.05). These results indicate that X-irradiation induces delayed aneuploidy of chromosomes 1 and 4 in fibroblasts.

## 4. Discussion

In this study, micronucleus-centromere assays using pancentromeric or centromeric probes of chromosomes 1 and 4 were performed to examine RIGI occurring in irradiated human fibroblasts as a function of time. Using a pancentromeric probe, MN formation through both chromosome loss and breakage can be identified by detecting the presence or absence of centromeric signals. Frequency of MN increased in a dose-dependent manner in irradiated cells at 28, 88 and 240 h. The micronucleus-centromere analysis using a pan-centromeric probe showed that X-irradiation-induced MN were predominantly MNC− (70%). This observation is clearly in agreement with the well-known clastogenic properties of ionizing radiation as shown in previous studies [36,37].

Our results showed no significant difference in MN frequency between the cells analyzed at 28 and 88 or 240 h. Furthermore, MNC− and MNC+ frequencies showed a tendency for decremented decreases and incremental increases over the cell divisions, respectively, although these changes were not statistically significant. MN can arise from either acentric fragments (MNC−) [38] or from whole chromosomes (predominantly, MNC+) [39]. In general, MN are expected to disappear over time because acentric fragments or unstable chromosomes are not transmissible to successive cell generations [40]. The persistence in total MN frequencies across generations observed in this study may be partially explained by this increase in MNC+ frequency with cell passage, although it was not significant. However, the effect did persist across generations when considering total MN as the sum of MNC+ and MNC−. It is also possible that the initial induction of MN after low-dose X-irradiation persists within the first 5 cell generations, but is then lost in subsequent generations.

In addition, several studies reported that DSBs are the initiators of RIGI [8,41] and DSB repair defects have been proposed as a possible mechanism of delayed genomic instability [4,42]. Suzuki *et al.* [8] reported that radiation-induced DNA DSBs could cause nonlethal, “potentially unstable chromosome regions” (PUCR), which are altered chromatin architectures within the nucleus through DNA repair. PUCR may be transmissible for many generations after irradiation and could be regions susceptible to delayed DNA breakages [43]. Recurring breaks at a PUCR-like region in our cell lines could be an explanation for the persistence of MN across generations in the cells at 240 h after irradiation.

RIGI is defined as an “elevation in the rate of *de novo* genetic alterations in the genome of the progeny multiple generations after the direct insult” [44,45]. The present study shows that aneuploidy frequency in chromosomes 1 and 4 increased with radiation dose in fibroblasts at 28, 88 and 240 h. Interestingly, a significantly higher aneuploidy of chromosomes 1 and 4 was observed in the cells analyzed at 240 h compared to the cells analyzed at 28 h. Aneuploidy is known to be formed due to malfunction of the mitotic spindles or damaged kinetochores [46]. Ford *et al.* [47] also reported that chromosomes that fail to attach to the spindle at metaphase lag behind at anaphase and then are either (1) sorted into the MN pool, forming MNC+ or (2) mal-segregated between the two daughter nuclei. Cells containing unstable, and initially induced aberrations after irradiation are lost at a rate of ~50% per cell division. However, our results showed a *de novo* appearance of chromosomal aberrations within five population doublings. These findings indicate that low-dose of X-irradiation is effective in inducing delayed aneuploidy of chromosomes 1 and 4 through non-disjunction and chromosome loss.

The mechanism involved in delayed aneuploidy in cells surviving X-ray is not fully understood. However, a study by Dahle and Kvam [48] can partially explain our results. They reported that X-rays increased the number of centrosomes in cell clones 14 days after irradiation. Centrosomes are known to have crucial functions in the correct segregation of chromosomes through microtubule formation during the mitotic cycle; additionally, cells with over-expressed centrosomes have disorganized spindle formation and variability in chromosome numbers [49]. Given the important role of centrosomes in mitotic spindle organization, it is possible that centrosomal aberrations after irradiation can result in delayed aneuploidy in the progeny of X-irradiated cells.

In the present study, X-irradiated cells were sub-cultured up to five passages at every population doubling level; however, it is possible that cells were lost during sub-cultures. Furthermore, we incubated the irradiated cells in cytochalasin-B for 28 h in order to evaluate the direct effect of X-ray irradiation. Considering the doubling time of the fibroblasts used in this study, our results likely do not include the effect of cells that were in G1 at irradiation. However, we tried different incubation time points with cytochalashin-B up to 40 h, and there was no significant difference in total MN frequency at different time points. Additional studies with high dose (>100 cGy) and/or an extended generations (beyond five population doublings) should also be performed in order to elucidate/better understand the delayed effect of low doses of radiation on genomic instability. However, our results provide useful information that can be used for policy makers, government regulators and agencies to set up strategies for limiting the dose of radiation exposure for both the public and radiation workers.

## 5. Conclusions

We examined whether low-dose of X-irradiation induces delayed chromosomal aberrations in normal human fibroblasts. The obtained results show that low-dose of X-ray can induce delayed aneuploidy of chromosomes 1 and 4 in the progeny of irradiated cells. Further investigations are still needed to determine the mechanism of radiation-induced instability; since understanding this mechanism is important for assessment of radiation risk and determining its potential role in radiation-induced carcinogenesis.
